# Independent Evaluation of Medical-Grade Bioresorbable Filaments for Fused Deposition Modelling/Fused Filament Fabrication of Tissue Engineered Constructs

**DOI:** 10.3390/polym10010040

**Published:** 2018-01-02

**Authors:** Mina Mohseni, Dietmar W. Hutmacher, Nathan J. Castro

**Affiliations:** Institute of Health and Biomedical Innovation, Queensland University of Technology, Brisbane City 4059, QLD, Australia; mina.mohseni@hdr.qut.edu.au (M.M.); dietmar.hutmacher@qut.edu.au (D.W.H.)

**Keywords:** tissue engineering and regenerative medicine, bioresorbable polymers, 3D printing/additive manufacturing, fused filament fabrication/fused deposition modelling, degradation, physicochemical characterization

## Abstract

Three-dimensional printing/additive manufacturing (3DP/AM) for tissue engineering and regenerative medicine (TE/RM) applications is a multifaceted research area encompassing biology, material science, engineering, and the clinical sciences. Although being quite mature as a research area, only a handful of clinical cases have been reported and even fewer commercial products have made it to the market. The regulatory pathway and costs associated with the introduction of bioresorbable materials for TE/RM have proven difficult to overcome, but greater access to 3DP/AM has spurred interest in the processing and availability of existing and new bioresorbable materials. For this purpose, herein, we introduce a series of medical-grade filaments for fused deposition modelling/fused filament fabrication (FDM/FFF) based on established and Federal Drug Administration (FDA)-approved polymers. Manufacturability, mechanical characterization, and accelerated degradation studies have been conducted to evaluate the suitability of each material for TE/RM applications. The comparative data serves to introduce these materials, as well as a benchmark to evaluate their potential in hard and soft tissue engineering from a physicochemical perspective.

## 1. Introduction

Three-dimensional printing (3DP) has established itself as a robust and effective technology for the manufacturing of highly controlled micro- and macro-scale structures that are suitable for use as tissue-engineered constructs (TECs). In combination with computer-aided drafting (CAD) and finite element modelling (FEM), custom TECs with tunable and pre-designed porous networks providing the necessary structural support that is required for defect repair and tissue regeneration can be realized. Rapid and precise fabrication of mechanically-analogous three-dimensional (3D) microenvironments make this technique most attractive. Not solely as a tool for controllable 3D architecture, but with the introduction and availability of high quality and traceable materials, spatiotemporal controlled delivery of biomolecular agents are feasible when compared to conventional manufacturing methods. Therefore, 3DP is a leading technology in both tissue engineering and drug delivery.

Fused-deposition modelling/fused filament fabrication (FDM/FFF) is one of the most common and accessible 3DP technologies. FDM/FFF is an additive manufacturing technology that is based on melt extrusion of thermoplastic polymers with ordered deposition in a layer-by-layer fashion. With increasing interest in 3DP as a direct manufacturing technology, global demands for synthetic and natural materials with 3DP processability is a fast growing and untapped market. Although a plethora of single material and composite filaments are commercially available, the regulatory quality assurance/quality controls necessary for implantable 3DP medical devices from thermoplastic precursor filaments have limited translation to the clinic with few entities in pursuit of this burgeoning market. Therefore, the climate is ripe for the introduction of medical-grade 3DP thermoplastic filaments materials requiring thorough characterization of their potential in tissue engineering and regenerative medicine (TE&RM) applications. Thermoplastic polyesters and polyethers are good candidate materials for 3DP, owing to their high tunability with respect to melt properties, a unique property that is not typically seen in natural polymers where heating results in aloss of bioactivity and structural integrity. A uniform viscose flow and proper cooling rates is associated with the melting temperature, purity, molecular weight, and degree of crystallinity are important factors that are necessary for high resolution and reproducible printing of FDM/FFF 3D TECs. 

TECs should exhibit physicochemical and mechanical properties suitable for initial tissue engraftment and maturation through the modulation of cell behavior, including mechanotransduction during TEC degradation. Cell-material interactions are complex where mechanical stimuli in the form of local deformation of binding receptors influence intracellular pathways leading to the promotion or inhibition of tissue growth [1,2]. Soft tissues, including adipose tissue, exhibit mechanoresponsiveness where adipogenesis is enhanced in the presence of static stretching, but impeded under static compression, as well as under dynamic loading [3]. In the case of bone TE&RM, bone deposition and resorption are mutually-exclusive events that are modulated by mechanical stimulation of the tissue [4,5]. According to the minimum effective strains (MES) hypothesis, applied strains greater than MES causes the adaptation of bone density and architectures, while strain below MES does not produce any change [6]. When considering the clinical application of 3D printed TECs, biomaterial selection and TEC architecture should be designed to withstand local deformation within the physiological range necessary for tissue deposition. The fundamental characteristics of the bulk material, the overall geometry of the TEC and the internal geometry synergistically regulate strain distribution on the TECs and, subsequently, the local deformation of microenvironments. 

In addition to the structural contributions of 3D printed TECs, physicochemical changes of the structure, and, ultimately, the material during degradation, is another important aspect when designing TECs for TE&RM. Ideally, the rate of degradation and strength loss should be proportional to the rate of tissue growth [7]. As a TEC degrades, a loss in mechanical properties ensues and the rate of this action is a necessary consideration when designing TECs that inherently experience high stress. The rate of degradation and subsequent strength loss is an important design parameter for TE&RM research. Structural and mechanical integrity should remain during tissue remodelling, especially for large volume applications. As previously stated, the function of a scaffold is not only as a mechanical support, but also to serve as a substrate for tissue engraftment and remodelling. 

Accelerated degradation of is a widely accepted method to study in vitro physiochemical changes of polymers [8]. The degradation rate of synthetic polyester and polyether polymers can vary from days-to-months, while accelerated degradation provides short-term degradation profiles, which makes it a time and cost effective method to evaluate and characterize materials in vitro [9,10,11]. In vivo degradation of large molecular chains predominantly begins and proceeds by the hydrolysis of amorphous regions and short chains in the polymer backbone, rather than through enzymatic degradation [12]. Utilizing alkaline medium, including sodium hydroxide (NaOH) solutions, which is rich in hydroxyl (–OH) groups, increases hydrolysis and serves as a comparable method for in vivo conditions [13]. 

In the current study, we have extensively characterized four new medical-grade filaments (Dioxaprene^®^ 100 M, Max-Prene^®^ 955, Lactoprene^®^ 100 M, Caproprene^TM^ 100 M kindly donated by Poly-Med, Inc., Anderson, SC, USA) for 3D printability. More importantly, the effects of degradation on physicochemical properties have been evaluated, with special emphasis on mechanical properties. We have assessed these materials for printability via FDM/FFF by optimizing material-specific printing parameters, as shown in Table 1, as well as undertaken a comprehensive study characterizing physicochemical changes of 3D printed structures via accelerated degradation in alkaline conditions. Thermal analyses were also conducted to elucidate melt and flow properties for the optimization of printing temperature. In addition to melt behavior, thermal analysis allows for a better understanding of the morphology of macromolecular chains and re-crystallization of absorbable polymers. We conclude with a discussion wherein appropriate applications for each group of material is suggested based on degradation rate and strength loss.

## 2. Materials and Methods

### 2.1. Materials

Dioxaprene^®^ 100 M (DIO), Caproprene^TM^ 100 M (CAP), Lactoprene^®^ 100 M (LAC), Max-Prene^®^ 955 (MAX) were kindly donated by Poly-Med, Inc. (Poly-Med, Inc., Anderson, SC, USA). All of the materials were stored in a low humidity cabinet to minimize moisture absorption. Sodium hydroxide (NaOH, *M*_w_ = 40.00, Sigma-Aldrich, St. Louis, MO, USA) solutions of 1 M and 5 M were prepared with ultrapure water and used in all experiments. 

### 2.2. Printing 3D Scaffolds

A series of 10 mm × 10 mm × 5 mm models were designed in SolidWorks (Dassault Systemes, Waltham, MA, USA) and the resultant computer-aided design (CAD) file was prepared for 3D printing by conversion to a computer numerical control file by Simplify3D^®^ (Blue Ash, OH, USA). Next, the models were printed using a FlashForge Dreamer table-top FDM/FFF printer (FlashForge, Jinhua, China) at varying infill densities of: 10%, 20%, and 40%, respectively. 

Infill density is a printing parameter that is related to the number of printed fibers comprising the internal architecture of the printed part. The porosity for each infill density was calculated as the ratio of actual volume to bulk volume using the following formula, where $V_{b}$ is the bulk volume and $V_{t}$ is the true volume.

(1) $$\rho =\frac{V_{b}-V_{t}}{V_{b}}\times 100$$

Actual volume was calculated as the mass ratio between the printed scaffold and bulk density of the material, as determined by the volume of the rectangular geometry (Table 2).

### 2.3. Degradation

Accelerated degradation was conducted for porous scaffolds using NaOH as the hydrolysis medium. A 1 M NaOH solution was used to study degradation for all four materials. Due to the slow degradation rate of PCL in 1 M NaOH, a higher concentration (5 M NaOH) was employed. The samples were immersed into NaOH and incubated at 37 °C, 5% CO_2_. At each respective time point, degraded samples were rinsed with deionized water (3×) and dried under vacuum at 40 °C for 8 h. Mass loss was calculated as the ratio of residual mass $(W_{r}$) and initial mass $(W_{0}$), according to the general formula.

(2) $$W_{L}\%=\frac{W_{0}-W_{r}}{W_{0}}\%$$

### 2.4. Scanning Electron Microscopy

The microstructure of pristine and degraded samples were analysed by scanning electron microscopy (SEM, JSM-7001F, JEOL Ltd., Tokyo, Japan). For cross-sectional views, samples were flash frozen in liquid nitrogen (5 min) and cut. All of the samples were gold sputter-coated using a JEOL fine sputter coater (JFC-1200, JEOL Ltd., Tokyo, Japan) for 75 s at 8 mA current and observed under vacuum at 2 KV accelerating voltage. Scaffold pore size was determined by measuring 10 fields of view using ImageJ (National Institutes of Health, Bethesda, MD, USA) [14]. 

### 2.5. Thermal Characterization

Melt and thermal properties of pristine and degraded materials were characterized via differential scanning calorimetry (Q100 DSC, TA Instruments, Newcastle, DE, USA) under non-isothermal conditions. Additionally, thermal transitions of non-printed monofilaments were determined to serve as a good estimation of the printing temperature. Samples weighing approximately 4 mg were sealed in an aluminium pan and exposed to non-isothermal heating at a ramp rate of 10 °C/min with kinetic analysis performed on the thermographs utilizing universal analysis 2000 software (TA Instruments, Newcastle, DE, USA). 

### 2.6. Mechanical Testing

Unconfined, uniaxial compression was conducted on pristine and degraded samples using an Instron Micro Tester (5848, Instron, Melbourne, Australia). In an effort to characterize the effects of material composition and pore size on the mechanical properties, 3D printed TECs with three porosities were printed and evaluated. To study the effects of degradation on TEC stiffness, mechanical analysis was conducted for degraded samples. TECs of 20% infill density were chosen for strength loss characterization. Briefly, specimens were placed on a flat platen and were compressed at a rate of 0.6 mm/min up to 50% compression (*n* = 5) in deionized water at 37 °C. The elastic modulus was defined as the slope of the linear region (Range 4–10%), with the yield strength being defined as the peak stress of the linear region. 

### 2.7. Contact Angle Analysis

Surface wettability of 3D printed TECs was evaluated by contact angle analysis. Thin films of the each polymer was prepared by heating to the respective melting point in an oven and cooled on a 20 mm circular glass cover slip at room temperature. A droplet with the volume of 2 μL was deposited on the films with images that were taken at the static condition using the FTA200 computer-controlled, video based instrument (First Ten Angstroms, Portsmouth, VA, USA). Five positions were randomly tested for each sample.

## 3. Results

### 3.1. Degradation

**Figure 1:**
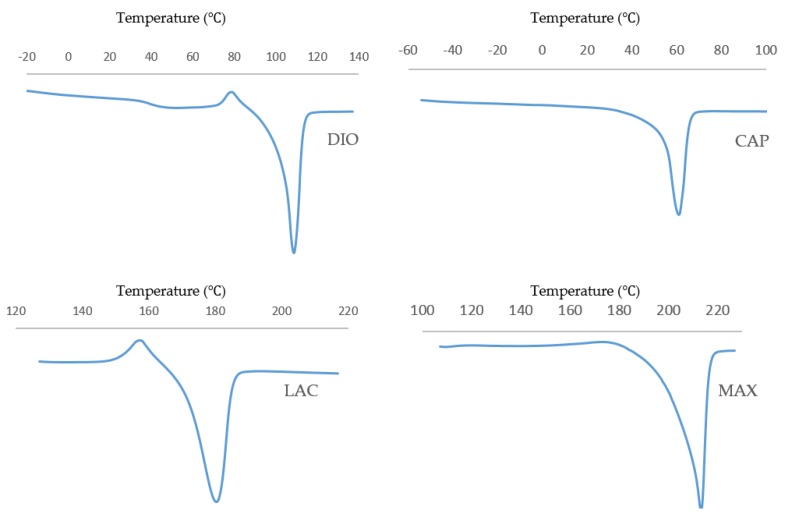
Non-isothermal DSC trace of non-printed monofilaments at a heating rate of 10 °C/min.

**Figure 2:**
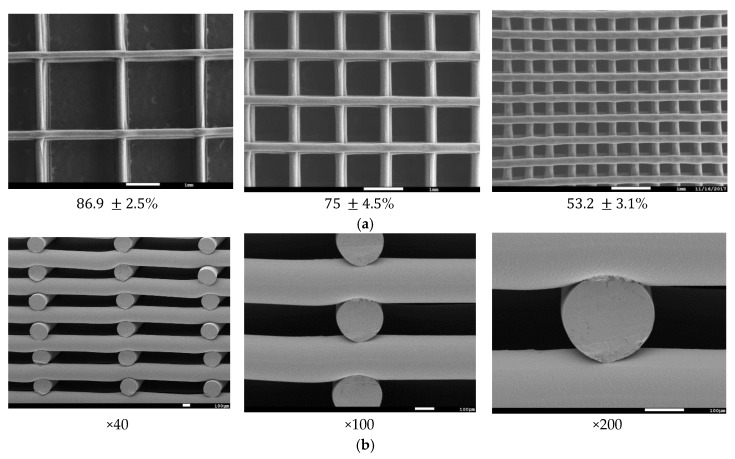
Scanning electron microscopy (SEM) micrographs of three-dimensional (3D) printed scaffolds; (**a**) Representation of three porosities; (**b**) Representation of cross-sectional view at varying magnification.

Thermal characterization of pristine, unprinted monofilaments by DSC (Figure 1) was used to determine the optimal temperature that was necessary for stable viscous flow and printability of the molten polymer. Table 1 shows optimized layer height, printing temperature, and material feed rate. Due to rapid cooling, interlayer fusion of MAX was inadequate at a layer height of 0.2 mm resulting in delamination. Therefore, a layer height of 0.16 mm was used. All of the materials were printed successfully, and SEM images were taken to quantify and validate the pore size of three different infill densities (Figure 2) with corresponding porosities, as determined using Equation (1).

Although DSC analysis is a good predictor of printing temperature, it is not reflective of the optimized printing temperature. For instance, PCL does not exhibit viscous flow up to 120 °C, while for all other materials, the printing temperature closely matches the melting point. This may be due to differences in the inherent viscosity and molecular weight of the material at the respective melt temperature.

### 3.2. Contact Angle Analysis

**Figure 3:**
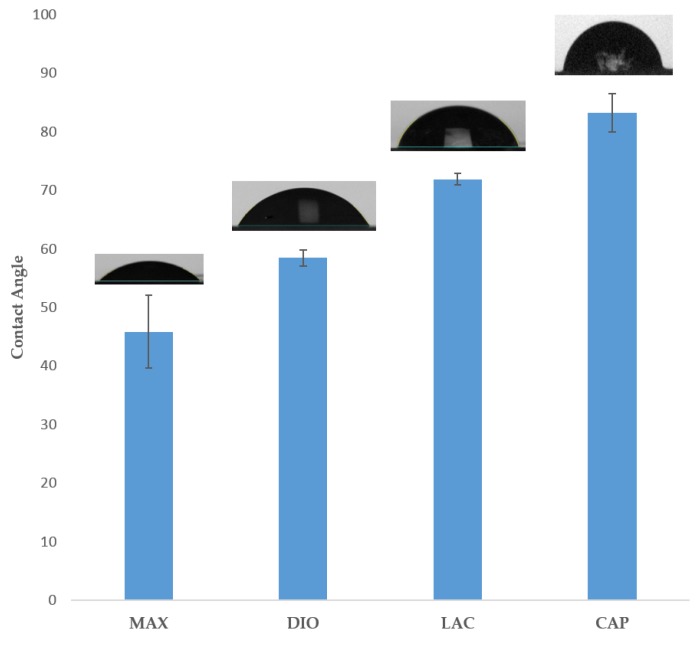
Water contact angle analysis of polymer films. Data is represented as mean ± SD (n=5).

Figure 3 illustrates surface wettability, as determined by contact angle analysis of the four materials. Results show MAX as the most hydrophilic with a contact angle of 45.8° ± 1.8°, with increasing hydrophobicity of 58.4° ± 0.42°, 77.8° ± 1°, and 83.2° ± 3.3° for DIO, LAC, and CAP, respectively.

### 3.3. Mechanical Characterization

**Figure 4:**
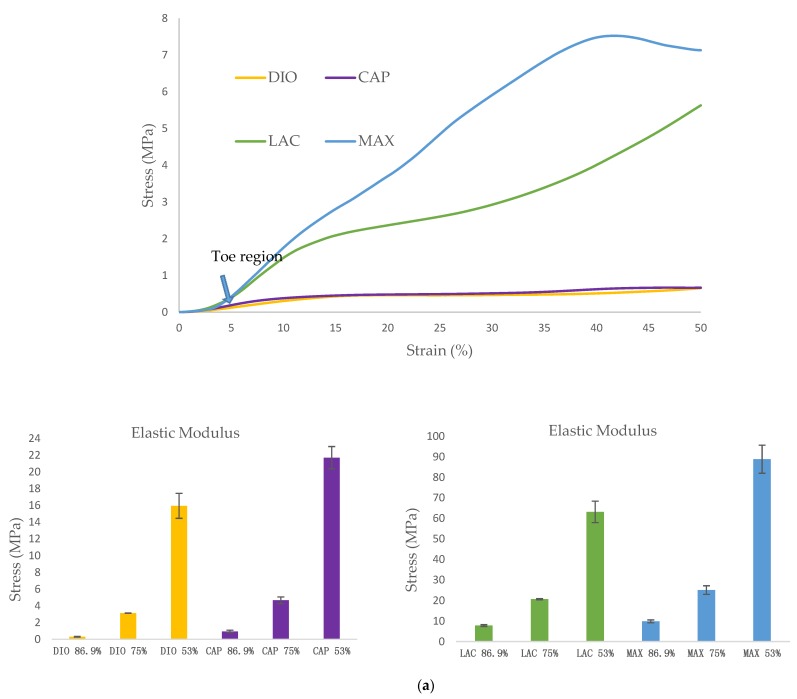

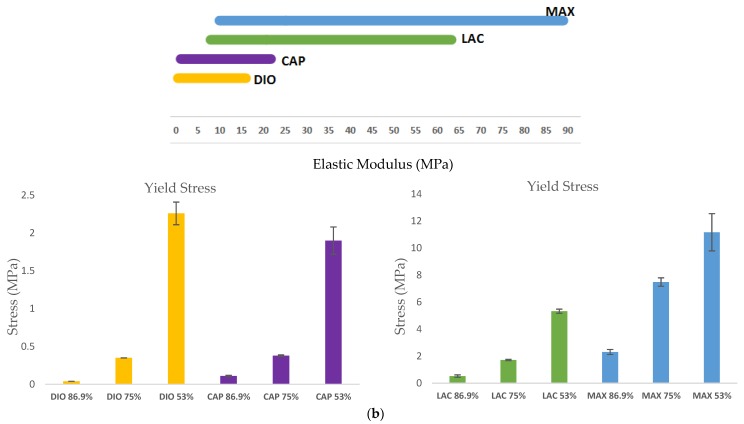
Unconfined, uniaxial compression. (**a**) Stress-strain curves for the scaffolds with different pore size; (**b**) Compressive modulus and yield strength for all materials with three porosities. Data is represented as mean ± SD (n=5).

The mechanical behavior of 3D scaffolds under compression was characterized and is shown in Figure 4. All of the materials exhibit a toe region followed by linear and subsequent plastic deformation (Figure 4a). It is observed that mechanical strength of 3D printed structures is dependent on the inherent properties of the material, as well as pore size. When comparing the four materials, DIO with a pore size of 2.19 mm is the softest exhibiting a compressive modulus of 0.3 MPa which is 3.1, 25.8, and 32.6-fold less than CAP, LAC, and MAX, respectively. An increase in porosity leads to a significant increase in compressive modulus and yield strength, with DIO exhibiting the greatest change with respect to pore size as an increase in porosity from 53.2% to 86.9% produces a 53-fold increase in elastic modulus while similar porosities produce a 23, 8, and 9-fold increase for CAP, LAC, and MAX, respectively (Figure 4b). The inherent properties of the material and regulating pore size through precise 3D printing make it possible to achieve the desired mechanical properties.

### 3.4. Degradation

**Figure 5:**
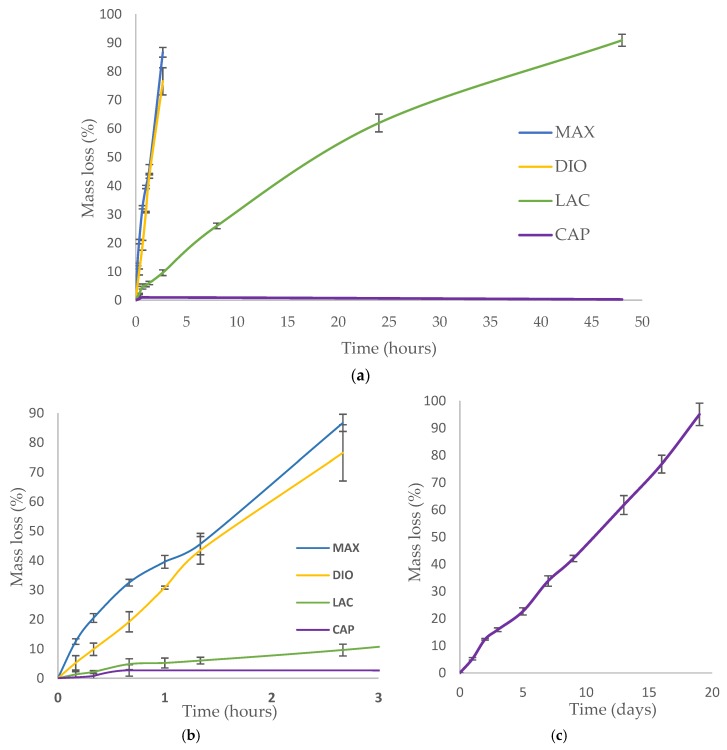
Accelerated degradation; (**a**,**b**) Degradation of MAX, DIO, LAC, and PCL in 1 M NaOH. (**c**) Degradation of CAP in 5 M NaOH. Data is represented as mean ± SD (n=5).

Accelerated degradation was conducted under alkaline conditions to quantify the rate of mass loss, strength loss, and to evaluate morphological changes of printed fibers. 1 M NaOH was used to accelerate hydrolytic degradation of DIO, LAC, and MAX. Since the degradation rate of CAP in 1 M NaOH is considerably slower than all of the other materials (Figure 5a, less than 1% after 24 h), 5 M NaOH was used to accelerate CAP degradation within a comparable time frame. Hydrolytic degradation of polymers occurs by erosion of the polymer beginning with cleavage of hydrolytic bonds leading to the formation of water soluble components [15]. All of the materials show non-uniform mass loss under accelerated degradation at the early stages, which is associated with the cleavage of amorphous regions within the polymer. The degradation rate of amorphous regions is typically higher than crystalline regions as the accessibility of water molecules is less energetically costly. Single crystals in close proximity to the degraded amorphous region become unstable leading to cleavage and solubility. As a result, crystalline regions become more accessible and susceptible to hydrolytic cleavage and erosion.

MAX and DIO exhibit the fastest degradation rate, more than 75% mass loss after 2 h, while LAC shows less than 10% degradation (Figure 5b). When comparing LAC and CAP, after two days, LAC exhibited >90% mass loss, while CAP exhibited <1% degradation. In 5 M NaOH CAP shows accelerated degradation leading to ~95% mass loss after 19 days (Figure 5c). 

**Figure 6:**
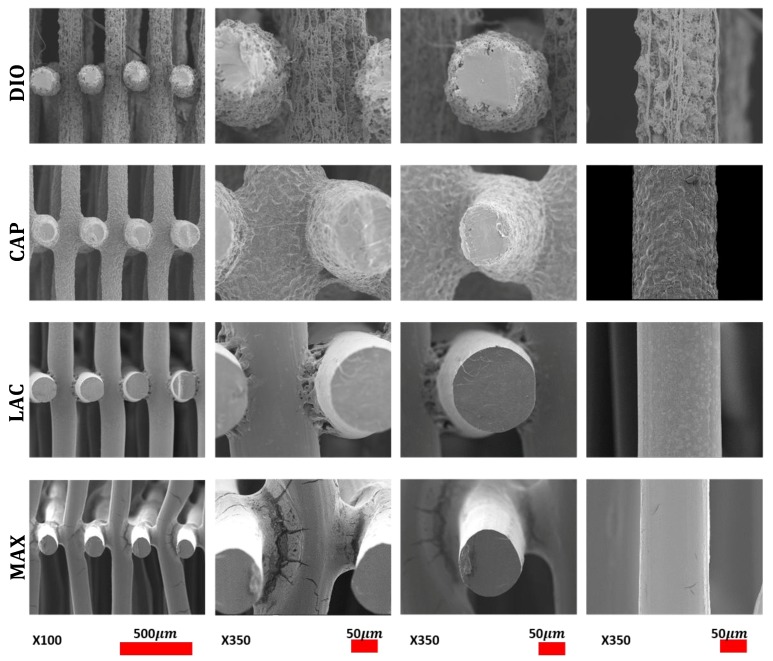
SEM micrographs of degraded samples after losing 30% mass loss at different magnifications.

Morphological studies were conducted via SEM of pristine and eroded samples. Erosion patterning and subsequent morphological changes of the printed structures are directly related to water accessibility and surface penetration. SEM images of MAX and LAC show a smooth surface at the top and cross section of the printed fibers, which indicates that the speed of water penetration into the core of the printed fiber is slower than the speed of hydrolysis of ester bonds. Subsequently, both amorphous and crystalline regions disintegrate prior to diffusion into the subsequent layers. A morphologically smooth surface and uniform decrease of fiber diameter and thickness leads to increased pore size (Figure 6 (DIO/CAP)). The results are indicative of a predominant surface erosion mechanism of degradation. For MAX, fusion points exhibit a similar erosion pattern with a regular smooth surface, while for LAC, fusion points have irregular surface morphology, producing a different morphological structure when compared to other regions of the 3D printed part. Therefore, the rate of hydrolysis at the fusion points of LAC is lower than water penetration. This observation indicates higher and larger crystalline structures that disintegrate in a slower rate when compared to amorphous sites leading voids and non-uniform surfaces. This can be related to the print speed and the overlaying of molten material wherein the material is cooled at a slower rate allowing for better recrystallization. MAX shows a uniform decrease of fusion points with circumferential cracks followed by longitudinal fractures (Figure 6 (LAC/MAX)). When compared to MAX, LAC exhibits a lower density of microfractures on the surface and fusion points, with only a few seen at the fusion points at 90% mass loss (Figure 6 (LAC)). CAP displays a roughened surface due to variations in hydrolysis speed of amorphous and crystalline regions. Crystallites exhibit greater resistance to hydrolysis and are morphologically noted as irregular grains on the surface. Initially, amorphous regions are cleaved, leading to higher crystallite density with more uniform degradation rate. Due to hydrophobic surfaces and high resistance of PCL toward water penetration, degradation proceeds slowly through cleavage of crystallites. The cross sectional view of printed fibers also displays a smooth surface absent of voids, which is indicative of a dominant surface erosion mechanism. Hydrolysis begins at the surface and proceeds very slowly due to high resistance of CAP to water penetration. The erosion pathway of the fusion points and surfaces are morphologically similar. DIO shows large pores on the surfaces and fusion points, which are attributed to fast hydrolysis and increased accessibility of water molecules into the interior of polymer. The cross section view shows that degradation begins from the edges causing non-uniform structures. Since no voids are seen in the core of the printed fiber, the degradation mechanism is predominately surface erosion with the fusion points exhibiting similar morphological changes when compared to other sites.

**Figure 7:**
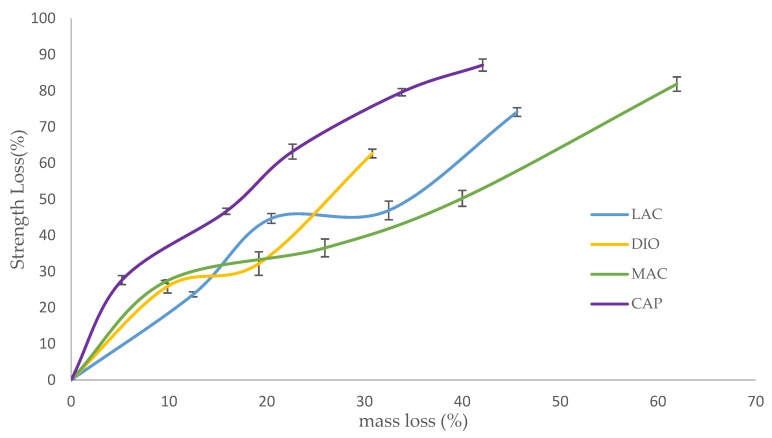
Strength loss versus mass loss for degraded scaffolds with porosity of 75%. Data is represented as mean ± SD (n=5).

Strength loss of degraded scaffolds is presented as a factor of mass loss in an effort to evaluate the materials’ potential for use in applications experiencing moderate to high levels of stress (Figure 7). For all of the samples, strength loss begins with a quick degradation rate followed by a rate decrease until complete disintegration. The rapid initial strength loss can be attributed to the initial attack and scission of amorphous regions by water molecules. This scission produces shortened polymer chains, which can be readily dissolved. At high mass loss, when the density of cracks is increased and the structure is susceptible to disintegration, the rate of strength loss increases again until complete fragmentation. When comparing the rates of mass loss to strength loss, the rate of strength loss exceeds that of mass loss due to the scission of amorphous regions and short chain polymers before the complete cleavage of the polymer chains ensues; mass loss appears as the chains are completely broken and dissolved in water. Before deviations in mass are detectable, strength loss is observed due to small scissions and cracks. MAX maintains structural and mechanical integrity up to 45% mass loss, while LAC preserves mechanical integrity up to 61% mass loss. Although these two polymers have a close mechanical behavior, the rate of strength loss of MAX is faster due to the higher density of cracks. Developed circumferential cracks at fusion points and longitudinal cracks on the surfaces increase stress concentration and the risk of failure. DIO shows an integrated structure of up to 30% mass loss, while PCL maintains its’ integrity up to 42% mass loss. DIO is more fragile when compared to PCL and collapses sooner. As it is observed from SEM analysis, DIO shows large pores at fusion points due to faster hydrolysis speed and higher accessibility of water molecules to the interior of DIO. Higher stress concentration at irregular edges and smaller contact area at fusion points make DIO more fragile.

### 3.5. Thermal Analysis

Thermal transition of 3D printed non-degraded and degraded materials was monitored under non-isothermal analysis at a heating rate of 10 °C/min to evaluate the changes in the morphology and crystallinity of materials. 

**Figure 8:**
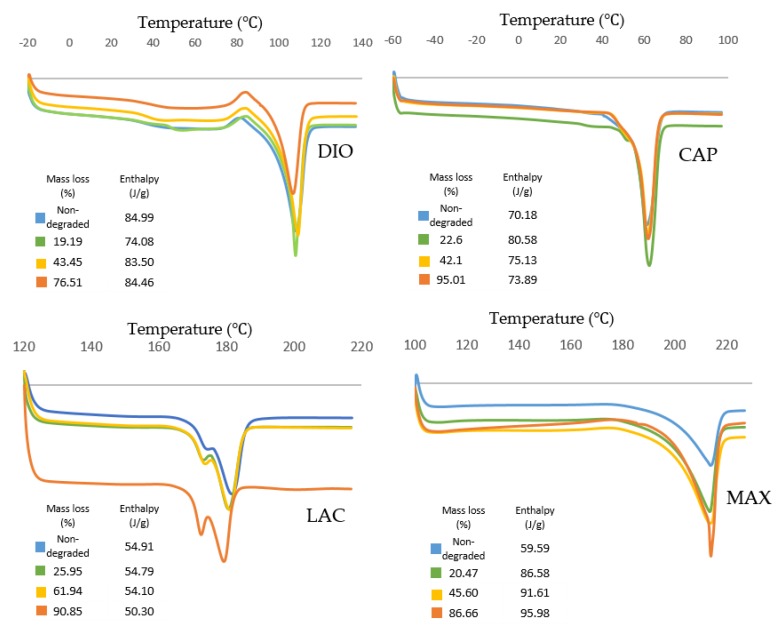
Thermal characteristics of non-degraded and degraded materials with different mass loss. Non-isothermal analysis was conducted at heating rate of 10 °C/min using DSC.

Thermal behavior of all materials is displayed in Figure 8. DIO shows an exothermic peak before melting which is related to the recrystallization of the polymer. For samples with higher degrees of degradation, the recrystallization temperature increases from 81.3 to 85.4 °C, which can be attributed to the larger lamellae requiring higher temperature and more energy for recrystallization. 

Thermal transition of CAP shows an increase of enthalpy for degraded samples when compared to non-degraded samples. This observation illustrates a higher degree of crystallinity for degraded samples resulting in a slower degradation rate. CAP shows the highest melting peak and also the highest crystallinity (80.58 J/g) at 23% mass loss, which is the maximum resistance of crystalline structures toward hydrolysis and cleavage.

Thermal analysis of LAC shows a double melting behavior that can be explained by the melt-recrystallization model where the first endothermic peak is attributed to the melting of original lamellar structures, while the second endothermic peak is associated to the melting of newly formed crystallites during recrystallization. As the material melts during the first endothermic peak $T_{m1}$, larger lamellas begin to recrystallize leading to energy release. The newly formed crystalline structures melt at the higher temperature, $T_{m2}$, resulting in the second endothermic peak. As degradation proceeds, $T_{m1}$ and $T_{m2}$ decreases producing smaller original lamellas with lower molecule weights. The enthalpy decreases gradually from 54.91 to 54.10 J/g as materials lose their mass up to 61.94%. At 90.85% mass loss, enthalpy has reduced significantly to 50.30 J/g.

For MAX, during degradation the area of endothermic peaks increases from 59.59 to 95.98 J/g showing an increase in crystallinity. Additionally, the endothermic peak of the sample with the highest mass loss exhibits a narrowing which shows less crystallite size distribution. A small exothermic peak is observed before the melting peak that increases during degradation. This exothermic peak can be attributed to recrystallization before melting. Since higher levels of degradation produce a higher degree of ordered structures, there is greater probability of recrystallization as indicated by increasing exothermic peaks.

## 4. Discussion

In the current study, we have evaluated four new medical grade filaments for printability, physicochemical characteristics, and the potential for use in TE&RM applications. Comprehensive characterization was performed on non-degraded and degraded materials to assess their physical, mechanical, and morphological properties before and after degradation. These studies provide greater insight and empirical evidence to support proposed TE&RM applications.

Optimized printing conditions were determined after evaluating the heating effects on phase transition of monofilaments via DSC (Table 1, Figure 1). The materials were printed successfully with three different porosities and were assessed under unconfined, uniaxial compression to quantify the compressive modulus and yield strength. DIO with a pore size of 2.19 mm shows the softest behavior with an elastic modulus of 300 KPa (Figure 4b). DIO is composed of 100% Polydioxanone (PDO), which is a synthetic resorbable polymer with high flexibility supported by ether oxygen bonds and used extensively as an implantable suture. It exhibits excellent biocompatibility, degradability, and high flexibility, making it appropriate for a variety of biomedical applications. DIO is very sensitive to internal structures and provides a wide range of mechanical properties by tuning the pore size of the printed TEC, which makes it highly desirable for TE&RM applications (Figure 4b). This study is the first reported use of DIO in FFF/FDM 3D printing showing high proccessibility. This potential along with the inherent soft behavior makes it a good candidate for complex structures in soft tissue engineering. CAP, which is 100% caprolactone, is another appropriate candidate for soft tissue engineering. When compared to DIO, it shows a higher elastic modulus, however, as the pore size decreases, the difference is less dramatic. At a pore size of 2.19 mm, elastic modulus of PCL is approximately three times higher than DIO, while at a pore size of 0.37 mm, it is 1.3-fold higher. MAX, a copolymer of 95% glycolide and 5% lactide, shows the highest elastic modulus, at the pore size of 0.37 mm. Changing pore size from 0.37 to 2.19 mm gives a wide range of properties from 9.8 to 88.9 MPa. LAC is 100% Lactide and shows various elastic modulus from 7.79 to 63.17 MPa. By controlling the pore size of MAX and LAC, a wide range of materials properties can be achieved, which are suitable for hard tissue engineering including cartilage and bone tissue.

Accelerated degradation in alkaline medium was conducted to investigate hydrolytic degradation of all the monofilaments. Hydrolysis is a chemical reaction of water molecules with polymers that produces carboxylic acid, followed by cleavage of -OH bonds, resulting in the erosion of polymer matrix [16]. In this study, mass loss rate was evaluated in NaOH and depicted in Figure 5. By comparison of these results with contact angle analysis, it is realized that more hydrophilic surfaces shows higher degradation speed (Figure 3). SEM analysis shows surface erosion for four materials as the predominant degradation pathway. Figure 6 illustrates that degradation begins from the edges with no voids being seen radially towards the core. However, surface morphology of each polymer is different due to various rates of hydrolysis and water penetration into the polymer fibre with DIO exhibiting large voids on the surfaces. Hydrophilic surfaces for DIO, as well as fast hydrolysis, results in decreased stability and porosity. PCL exhibits a roughened surface due to the presence of highly water-resistant crystallites. MAX and LAC show smooth surfaces and uniform edges, with MAX exhibiting a high density of cracks at the fusion points and fibre surface leading to decreased mechanical stability and greater water accessibility.

In designing TECs, the bulk properties of materials as well as their degradation behavior and physicochemical changes should be considered. The inherent properties of materials and the internal structures of scaffolds, including pore size, are predominant factors in controlling the mechanical properties of designed TECs. Depending on tissue morphology and the cascade of biological phenomena during the regenerative process, an “optimized” pore size can vary. Feng. B showed that pore size <400 μm limits the growth and infiltration of blood vessels in a porous bioceramic scaffolds [17]. JP. Temple studied osteogenesis and angiogenesis of large craniomaxillofacial bone defects, with TEC exhibiting pore sizes of 0.1–2 mm [18]. The results show that uniform distribution of human adipose-derived stem cells with high density of vascular network was obtained at a pore size of ~800 μm. For larger pore sizes, cell aggregation ensues, leading to sedimentation and decreased activity, whereas for the smaller pore sizes, cell aggregation is minimized. In contrast to large bone defects, for barrier membrane in dental applications, smaller pores (<200 μm) is suggested to control the growth and infiltration of epithelial and gingival fibroblasts at early stages [19]. By decreasing the rate of cellular infiltration at early time points, bone tissue has more space to mature and remodel over time. Infection is one of the major concerns for applications involving barrier membranes. By employing TECs with small pore sizes, the risk of infection can be minimized [19]. By considering the limitation of pore size in a specific application, materials and internal structures should be chosen carefully to ensure suitable conditions for growth of tissue.

Based on the current study, MAX and LAC may be suitable candidates for musculoskeletal tissue engineering. These materials exhibit excellent printing fidelity allowing for the manufacture of complex geometries for large and small defects. By controlling pore size, a wide range of properties can be achieved. In addition, by printing composite structures of MAX and LAC, the unique properties of each material can be combined with predefined geometries leading to a higher degree of control on mechanical properties as well as degradation rate. Depending on the volume of defects and the speed of regeneration, the degradability of composite structures can be tuned. MAX degrades within 36 h in alkaline solution, while LAC exhibits 90% mass loss after 48 h. A composite structure of these two materials with different degradation rates is also feasible and merits further investigation in particular for drug delivery applications, where the release profile of drugs can be controlled by faster degrading material and the structure of scaffolds can be preserved by long-term degradable materials. As previously stated, infection is one of the major concerns with implantable TECs, which can be addressed by controlled release of antimicrobial drugs within the first few days of implantation when macrophages show the highest activity [20]. Utilizing dual extruding printers and controlling the number of layers and internal structures of each material shows great promise for controlled mechanical properties, degradation, and drug release.

With regards to soft tissue engineering, most available printing materials are natural hydrogels, which cannot be readily printed in complex geometries, especially for large volume tissue regeneration [21]. In this study, DIO has been introduced as a soft polymer with high flexibility along with high 3D printing fidelity for complex small and large structures. When compared to CAP, DIO exhibits a faster degradation rate, which makes it more appropriate for short-term regenerative applications. DIO may also be an appropriate polymer matrix for artificial skin patches owing to its mechanical and physicochemical properties. Additionally, skin does not require extended degradation for long-term regeneration and remodelling, which makes DIO a better candidate when compared to CAP or other materials. Degradation of PCL, which is the main component of CAP, is very slow (>2 years) [9,22], and a combination of DIO and CAP may provide the added capability of adjusting the degradation rate of TECs to be temporally compatible with tissue regeneration and remodelling. In addition to dual extrusion FDM/FFF printing, these polymers can be mixed during printing as they have similar printing temperature. The potential addition of DIO into the TEC allows for greater tunability with regards to degradation rate and mechanical properties. Recently, the concept of harnessing the body as a “bioreactor” has been investigated by our group and coupled with delayed fat injection [23] has shown promising results for large volumes. Delaying the administration of fat into the TEC leverages the initial cascade of biological events leading to fibrous tissue infiltration and neovascularization. By controlling the degradation rate of the internal TEC architecture, adequate space post-implantation can be modulated allowing for more uniform tissue ingrowth. One potential embodiment would utilize DIO as the internal structure, while providing the mechanical behavior that is necessary to ensure tissue viability by using PCL as a slow degradable polymer. Another important positive aspect of composite structures is in drug delivery where the degradation rate directly affects the release rate of incorporated drugs. The hydrophobic surface of CAP, as well as its slow degradation, limits sustained delivery. In most therapeutic/regenerative applications, the speed of tissue regeneration is slow, while drug delivery is required for a short period post-implantation. Therefore, for combinatorial TECs, including antibacterial embodiments, antimicrobial agents need to be released at the early stages of regeneration. Releasing antibiotics within the first weeks prior to complete formation of blood clot can decrease the risk of infection considerably. Therefore, employing DIO as a delivery system in soft tissue engineering is suggested.

## 5. Conclusions

The current study served to introduce a family of 3D printable bioresorbable medical-grade materials for use in TE/RM applications. With the combination of material traceability and accessible high-resolution 3D printing technologies, we may find ourselves at the forefront of commercially-viable 3DP/AM for TE/RM applications.

## Figures and Tables

**Table 1:** Optimized printing parameters.

Material	Temperature (°C)	Feed-Rate (mm/min)	Layer Height (mm)
DIO	120	1800	0.2
CAP	120	1800	0.2
LAC	192	1350	0.2
MAX	210	1800	0.16

**Table 2:**
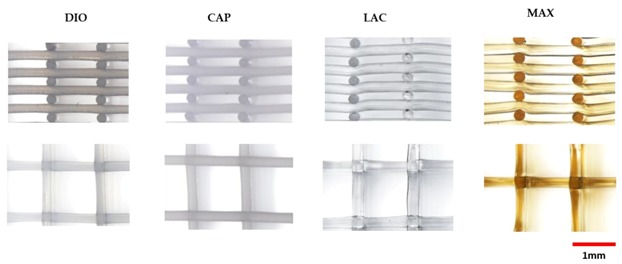
The porosity and pore size for three infill densities. Data is represented as mean ± SD (n=5).

Infill Density (%)	Porosity (%)	Pore Size (mm)
10	86.9±2.5	2.19±0.05
20	75±4.5	1.01±0.02
40	53.2±3.1	0.37±0.02
